# Development and Characterization of Zein/Ag-Sr Doped Mesoporous Bioactive Glass Nanoparticles Coatings for Biomedical Applications

**DOI:** 10.3390/bioengineering9080367

**Published:** 2022-08-04

**Authors:** Syeda Ammara Batool, Ushna Liaquat, Iftikhar Ahmad Channa, Sadaf Jamal Gilani, Muhammad Atif Makhdoom, Muhammad Yasir, Jaweria Ashfaq, May Nasser bin Jumah, Muhammad Atiq ur Rehman

**Affiliations:** 1 Department of Materials Science and Engineering, Institute of Space Technology Islamabad, Islamabad 44000, Pakistan; 2 Thin Film Laboratory, Department of Metallurgical Engineering, NED University of Engineering and Technology, Off University Road, Karachi 75270, Pakistan; 3 Department of Basic Health Sciences, Preparatory Year, Princess Nourah Bint Abdulrahman University, Riyadh 11671, Saudi Arabia; 4 Institute of Metallurgy and Materials Engineering, University of the Punjab, Lahore 54590, Pakistan; 5 Biology Department, College of Science, Princess Nourah Bint Abdulrahman University, Riyadh 11671, Saudi Arabia; 6 Environment and Biomaterial Unit, Health Sciences Research Center, Princess Nourah Bint Abdulrahman University, Riyadh 11671, Saudi Arabia; 7 Saudi Society for Applied Science, Princess Nourah Bint Abdulrahman University, Riyadh 11671, Saudi Arabia

**Keywords:** antibacterial, biomedical implants, electrophoretic deposition, osteogenesis

## Abstract

Implants are used to replace damaged biological structures in human body. Although stainless steel (SS) is a well-known implant material, corrosion of SS implants leads to the release of toxic metallic ions, which produce harmful effects in human body. To prevent material degradation and its harmful repercussions, these implanted materials are subjected to biocompatible coatings. Polymeric coatings play a vital role in enhancing the mechanical and biological integrity of the implanted devices. Zein is a natural protein extracted from corn and is known to have good biocompatibility and biodegradability. In this study, zein/Ag-Sr doped mesoporous bioactive glass nanoparticles (Ag-Sr MBGNs) were deposited on SS substrates via electrophoretic deposition (EPD) at different parameters. Ag and Sr ions were added to impart antibacterial and osteogenic properties to the coatings, respectively. In order to examine the surface morphology of coatings, optical microscopy and scanning electron microscopy (SEM) were performed. To analyze mechanical strength, a pencil scratch test, bend test, and corrosion and wear tests were conducted on zein/Ag-Sr doped MBGN coatings. The results show good adhesion strength, wettability, corrosion, and wear resistance for zein/Ag-Sr doped MBGN coatings as compared to bare SS substrate. Thus, good mechanical and biological properties were observed for zein/Ag-Sr doped MBGN coatings. Results suggested these zein/Ag-Sr MBGNs coatings have great potential in bone regeneration applications.

## 1. Introduction

Biomaterials are materials that can reside in a biological system to perform a certain function without inducing any toxic effect. They have a wide range of applications in bioengineering, pharmaceutics, biomedical implants, etc. The global biomedical implants market was reported to have a worth of ~USD 86 billion in 2019 and was anticipated to reach ~USD 147 billion by 2027, with 7.2% growth in the period 2020–2027. Growth in demographic aging and dire medical conditions influence recent technological progression in the bioimplants market [1]. The consistent growth of the implants market brought the interest of many researchers to this field. Metals, ceramics, polymers, and composites are potentially being used in the fabrication of different types of implants, e.g., cardiovascular, orthopedic, cochlear, dental, etc. Among all the suggested materials, metallic implants are broadly used in the fabrication of implant devices because, apart from being compatible in a biological system, they possess superior mechanical strength as compared to other materials [2]. Foreign bodies trigger the immune system to respond, which may result in fibrous encapsulation of a foreign body to isolate its interaction with the biological system [3]. For successful implantation, the implanted material should be familiar to the immune system so that the latter will respond positively.

Among all metallic implants, stainless steel (SS) implants have the highest proportion in the fabrication of orthopedic implants due to their cost effectiveness and accessibility. Poor corrosion resistance and relatively higher yield strength of SS implants in a physiological environment are the major drawbacks [4,5]. These implants, when interacting with a physiological environment, exhibit either corrosion or no response. Upon corrosion, SS implants release toxic metal ions (Ni and Cr ions) responsible for infections [6,7]. Corrosion impairs the function of implants by decreasing cell adhesion and mechanical strength, which ultimately leads to implant loosening [8]. The surface coating of SS implants with bio-composites can enhance corrosion resistance and bioactivity of the implants [4,9].

Biomaterial coatings on metallic implants improve the mechanical as well as biological properties of the implanted devices [10]. These coated materials can either be natural or synthetic as per the required properties of the final product. After implantation, the first component that interacts with the implant surface is water, followed by proteins. Thus, it is important to tune the surface of the implant to improve the initial protein attachment [11,12]. Zein is an alcohol soluble protein that largely contains prolamin. Zein was approved by the FDA in 1985 as a “generally recognized as safe” excipient. Zein is a natural polymer found in the endosperm cells of corn [13,14]. Zein contains hydrophobic, neutral amino acids (such as leucine, proline, and alanine), and some polar amino acid residues (glutamine). Zein outperforms other proteins because of the complete absence of lysine and tryptophan. The solubility of zein is restricted to acetone, acetic acid, aqueous alcohols, and aqueous alkaline solutions due to the amino acids [15]. Biopolymers are usually used with bioceramics due to their low mechanical strength, such as hydroxyapatite (HA) and bioactive glasses (BGs) [16]. BGs are third generation biomaterials widely used in bone tissue engineering applications. BGs can form strong bonds with the natural bone, thus they are considered as bioactive ceramics, which are usually used with other polymers [17,18,19]. Mesoporous bioactive glass nanoparticles (MBGNs) have small pores (2–7 nm) in them that increase the surface area of particles, which largely promotes its reactivity. MBGNs can be loaded with different drugs or metallic ions to facilitate bone tissue engineering, targeted drug delivery, and wound healing [20,21]. Tabia et al. [22] doped sol-gel derived MBGNs with magnesium and loaded them with amoxicillin. Results showed controlled drug release and high bioactivity due to the mesoporous structure and composition of BGs. Similarly, Ag and Sr ion doped MBGNs were prepared by [23] using a modified Stöber process. The presence of Ag ions imparts antibacterial properties, and Sr ions enhance the osteogenesis. An osteogenic material promotes bone regeneration and reduce bone resorption. Various coating techniques are being used to coat bioactive glasses for surface modification, such as thermal spraying [24], plasma spraying [25,26], radio frequency sputtering (RFS) [27,28], physical vapor deposition (PVD) [29], and electrophoretic deposition (EPD) [30,31]. EPD is well known for its applications in biomedical coatings [30]. EPD involves the movement and deposition of charged particles from the colloidal suspension in the presence of an electric field. EPD is a cost-effective process conducted at room temperature [32]. Numerous studies have been conducted on zein/bioactive glasses composite coatings deposited via EPD [32,33], but deposition of zein/MBGNs doped with metallic ions via EPD is a new approach to study the synergetic effect of dual ions. Evidently, we are the first to present a work on the development of zein/Ag-Sr doped MBGN coatings deposited on 316L SS via EPD.

In this study, we developed zein/Ag-Sr doped MBGN coatings on 316L SS substrates via EPD. After coatings were developed with designated parameters, coated substrates were subjected to material characterization. Optical microscopy and scanning electron microscopy (SEM) images verified the uniform deposition on 316L SS substrates. The coating showed antibacterial and bioactivity potential for biomedical applications.

## 2. Materials and Methods

### 2.1. Materials

Zein powder (CAS: 9010-66-6), absolute ethanol, and acetic acid were all purchased from Sigma-Aldrich^®^ (Taufkirchen, Germany). AISI 316L stainless steel (addressed 316L SS in this article) foil of ~1 mm thickness was used to prepare substrates. The composition of 316L SS was Ni-14.15, Cr-17.75, Mo-2.72, Mn-1.87, Si-0.58, P-0.015, S-0.008, C-0.025, Fe-balance (wt.%). Ag-Sr doped MBGNs were synthesized via a modified Stöber process; the details are given in our previous work [23].

### 2.2. Suspension Preparation

Prior to the EPD process, a stable suspension of zein/Ag-Sr doped MBGNs was made by following the study conducted by Rivera et al. [33,34]. Zein powder (6 wt.%) was added in a 100 mL beaker, followed by the addition of distilled water (20 wt.%) and absolute ethanol (74 wt.%). The mixture was stirred for 30 min at 35–40 °C on a magnetic hot plate, followed by another 30 min round of stirring at room temperature. Acetic acid (~10 mL) was added dropwise to maintain the pH value (~3.0) of the solution. Subsequently, Ag-Sr doped MBGNs (3 g/L) were added to the zein solution, followed by 30 min of magnetic stirring. The suspension was finally ultrasonicated for approximately 60 min to produce a stable suspension of zein/Ag-Sr doped MBGNs required for the EPD process. The concentration of Ag-Sr doped MBGNs was chosen on the basis of initial studies, which showed that the concentrations higher than 3 g/L results in the non-uniform and thick coatings. If the concentration of Ag-Sr doped MBGNs is less than 3 g/L, it would not be enough to impart bioactivity and antibacterial activity.

The stability of the suspension was determined by measuring the zeta potential of the pure zein, Ag-Sr doped MBGNs, and zein/Ag-Sr doped MBGNs suspensions in ethanol. A zetasizer (Malvern Instruments, Malvern, UK) was utilized for this purpose. The measurement was taken in triplicate for each suspension, and the average value with standard deviation is reported here.

### 2.3. EPD Process

The 316L SS foil was cut into pieces of 30 × 25 mm^2^ size. Subsequently, the pieces were cleaned in a mixture of acetone and absolute ethanol. It is important to note that no surface treatment was carried out on 316L SS foil. Substrates were rinsed with distilled water and dried at room temperature. The 316L SS was used as both working and counter electrodes at 10 mm inter-electrode spacing. Both electrodes were then submerged (half of total area) in 50 mL of initially prepared zein/Ag-Sr doped MBGN suspension. The coatings were developed by applying direct current (DC) on the substrates at different EPD parameters. EPD parameters were chosen on the basis of previous literature, which showed that lower deposition voltages (<10 V) led to the inhomogeneous coatings. In contrast to this, higher deposition voltages (>25 V) led to pronounced hydrolysis. The range 10–25 V of deposition voltages was investigated in the current study. The deposition time was kept fixed at 180 s on the basis of our previous studies [16].

### 2.4. Materials Characterization

The morphology and chemical composition of synthesized Ag-Sr doped MBGNs were examined via a scanning electron microscope (SEM-MIRA, TESCAN) equipped with energy dispersive spectroscopy (EDS).

Samples coated at different parameters were examined under an optical microscope (Novex) and SEM to study the morphology and thickness of the coatings. The samples were cut into 5 × 5 mm^2^ size prior to the examination.

A pencil scratch test (ASTM D3363-20) was conducted to measure the hardness of coatings. This test was performed manually by using graphite pencils of different hardness grade (8B to 2H). Scratches were made by the pencils held at an angle of 45° between the lead tip and the surface of the coated substrates. Starting from most hard (2H) to the softest grade (8B), pencils were pushed under uniform pressure to create a scratch throughout the sample width. The scratches were made until the pencil could not scratch the surface further. The lowest hardness grade of the pencil that is able to scratch the coating is considered the hardness of the coating. A bend test (ASTM-B571-97) was performed to check the adhesion/bending strength of the coatings. In order to perform the bend test, the coated surface was bent at 180° with the help of tweezers. After the bending test, the bending sites were examined to check for defects, i.e., cracks and delamination.

Contact angle measurements were done to determine surface wettability. A fixed volume (5 µL) of distilled water was dropped via microliter pipette on the surface of coated substrates. Digital images of the droplet were captured within 5 s of dropping, and the contact angles between the droplet and the surface of the coatings were measured by using Image J™. The test was performed at five different spots on the same coating, and the values of contact angles were averaged out. The mean values of contact angles were plotted with their standard deviations.

A tribometer (MT/60/NI, Spain) was used to conduct wear tests on the coated substrates. The ball-on-disk method was used, in which a steel ball indenter with a fixed load of 1 N was rotated for a distance of 50 m over the surface of the clamped substrates at 32 rpm. A graph between friction coefficient (COF; µ) and partial distance (m) was drawn to understand the wear behavior of the coating.

Corrosion tests were conducted using a three-electrode system Gamry Instrument (potentiostat reference 600, USA). A potentiodynamic polarization scan was recorded in simulated body fluid (SBF). The SBF was prepared according to the protocol reported in [35]. The composition of SBF is also noted in [35]. The coated sample was mounted as the working electrode with a graphite counter electrode. Ag-AgCl was used as the reference electrode. The test was conducted between the potential range of −0.5 V and +0.5 V at the scan rate of ~1 mV/s. The uncoated side of the substrate was covered with epoxy and dried prior to the test in order to make sure that corrosion results were obtained only from the selected surface area (1 cm^2^) of the coating. Before conducting this test, open circuit potential (OCP) was measured for an hour. A Tafel plot was constructed by extrapolating the cathodic and anodic branches. The corrosion potential (E_corr_) and corrosion current density (I_corr_) were measured for coated and uncoated samples.

### 2.5. Biological Characterization

The biological behavior of the deposited coatings was assessed via antibacterial and bioactivity analyses. Antibacterial tests were carried out against Gram-positive (*Staphylococcus aureus*; *S. aureus*) and Gram-negative (*Escherichia coli*; *E. coli*) bacterial strains. The coated samples were placed on agar plates containing 20 µL spread of each bacterial dilution. The samples were placed facing downwards to facilitate contact between coating and bacterial spread. The plates were then incubated at 37 °C. After 24 h, the inhibition zone on each plate was observed.

In vitro bioactivity test was carried out using SBF. Coated samples were placed in SBF (30 mL) and incubated in an orbital shaker at 37 °C for 1, 3, 5, and 7 days. The SBF was refreshed at alternate days to maintain the ionic concentration around the immersed samples. The coatings were removed at the designated time from the SBF, washed with de-ionized water, and air dried. The presence of an HA layer on the coated surface was confirmed via SEM/EDS analysis.

## 3. Results and Discussion

### 3.1. EPD Kinetics and Suspension Stability

Zein polymer is insoluble in water and organic solvents. It can be dissolved after inducing charge on its functional groups with the addition of acidic or basic medium. The polar and nonpolar groups present in zein are protonated in highly pure ethanol (<99%). The next step is to make a stable suspension of zein in ethanol. The stability of the suspension is analyzed using zeta potential measurements. The zeta potential of pure zein, Ag-Sr doped MBGNs, and zein/Ag-Sr doped MBGN suspensions were measured as +25 ± 10 mV, +16 ± 4 mV, and +22 ± 6 mV, respectively. According to the literature, polymer suspension should be sufficiently charged (positively or negatively) for effective electrophoretic mobility [36]. Particles with very low zeta potential tend to agglomerate and settle down due to strong attractive forces of interaction, whereas the particles with high values of zeta potential exhibit strong repulsive forces that inhibit the mobility of particles. In case of zein/Ag-Sr doped MBGN suspension, overall zein and Ag-Sr doped MBGNs are positively charged. Polymeric chains of zein contain some carboxylic side groups that are negatively charged, hence, the zein polymer wraps around the Ag-Sr doped MBGNs due to the attractive forces between oppositely charged side groups [37]. However, resultant zeta potential is positive, and zein molecules along with Ag-Sr doped MBGNs move towards the cathode and get deposited there following a mechanism termed charge stabilization [38], explained later in the kinetics of EPD.

Due to the addition of highly concentrated ethanol (>99%), the zein molecules get protonated and move towards the negatively charged electrode, hence cathodic deposition occurs. Hydrophilic groups of zein make bonds with Ag-Sr doped MBGNs and are deposited as a single entity. When the electric field is applied, water decomposes into H^+^ and OH^−^ ions in the presence of ethanol. The H^+^ ions towards the cathode due to their opposite charge and hydrogen gas evolves at the cathode, whereas the OH^−^ gets attached to the positively charged zein/Ag-Sr doped MBGNs in the suspension. The pH here increases due to the presence of OH^−^ ions. Overall, positively charged zein/Ag-Sr doped MBGNs get attracted towards the cathode, and, along with the OH^−^ ions, become unstable and get deposited on the cathode [39].

### 3.2. Morphology of Synthesized Ag-Sr Doped MBGNs and Coatings

**Figure 1:**
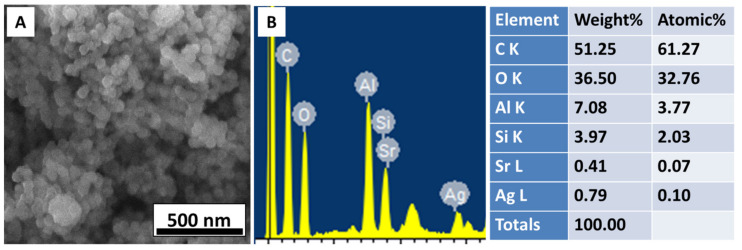
(**A**) SEM image showing spherical morphology of Ag-Sr doped MBGNs, (**B**) EDS spectrum confirms the doping of Ag and Sr in the bioactive glass network.

Figure 1A shows the morphology of the as-synthesized Ag-Sr doped MBGNs. The image confirms that the particles were spherical in shape, and the average diameter was approximately 92 nm. The Ag and Sr were detected in the EDS of the particles, as shown in Figure 1B.

**Figure 2:**
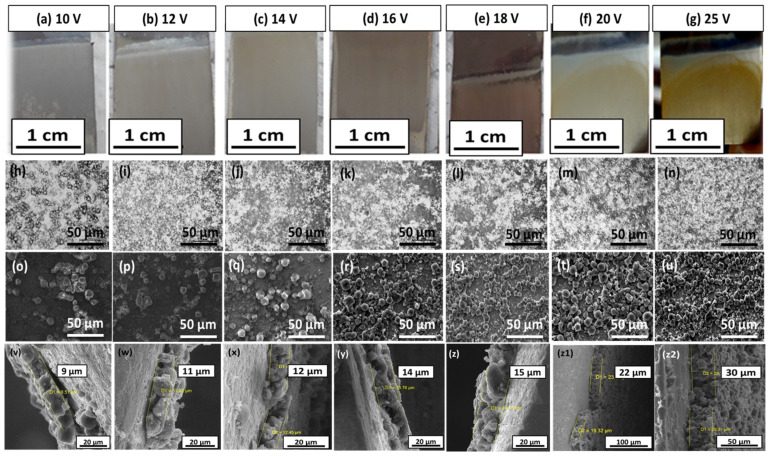
Digital images of zein/Ag-Sr doped MBGN coatings deposited on SS at (**a**) 10 V, (**b**) 12 V, (**c**) 14 V, (**d**) 16 V, (**e**) 18 V, (**f**) 20 V, and (**g**) 25 V. Optical and SEM images of coatings deposited at 10 V (**h**,**o**), 12 V (**i**,**p**), 14 V (**j**,**q**) and 16 V (**k**,**r**) showed non-uniform and less dense coating on SS, respectively. The coatings deposited at 18 V (**l**,**s**), 20 V (**m**,**t**), and 25 V (**n**,**u**) were more uniform and densely packed. The cross-sectional images show gradual increase in the thickness of coatings along with the voltage (**v**–**z2**).

Figure 2 shows the digital (a–g), optical (h–n), and SEM images (o–u) of zein/Ag-Sr doped MBGNs coatings developed on SS at different voltages. During the coating process, a slight change in the color of the coatings was observed at 18, 20, and 25 V (Figure 2e–g). Hydrolysis of suspension seems to be the reason for change in the color of coatings at higher voltages. Hydrolysis causes the chemical breakdown of materials by water. At higher voltages, hydrolysis increases, which causes the temperature of the suspension to rise. This increase in temperature may lead to the slight change in the color of coatings developed on SS substrates [40].

Further morphological analysis of zein/Ag-Sr doped MBGN coatings was done via optical microscope and SEM. Figure 2h–n show the images of zein/Ag-Sr doped MBGN composite coatings deposited at different parameters taken from an optical microscope. Optical micrographs showed uniform dispersion of composite coatings on 316L SS at all the deposition voltages. However, the densest coating was obtained at 25 V/180 s (Figure 2n) due to high deposition rate at a higher value of applied electric field. A similar trend was observed in [16], where the increase in applied electric field led to the increase in deposition yield and density of the coatings. The results of the current study are in agreement with Hamaker’s law [41].

Figure 2o–u show the SEM micrographs of zein/Ag-Sr doped MBGN coatings at different voltages. Figure 2o,p show that Ag-Sr doped MBGNs deposited at 10 V and 12 V were dispersed non-homogenously. A slight increase in voltage up to 14 V and 16 V (Figure 2q,r) shows large number of zein/Ag-Sr doped MBGNs deposited on SS with enhanced uniformity. At higher voltages of 18, 20, and 25 V, micrographs (Figure 2s–u) show the highly uniform and homogeneous dispersion of Ag-Sr doped MBGNs within the zein matrix, and the coatings were more densely packed. Large spherical agglomerates were formed during the electrophoretic deposition process [42]. The thickness of all coatings was measured from the cross section. It was observed that the thickness gradually increased along with the voltage. Figure 2v–z2 show the thickness of coatings deposited at various voltages. The coatings of 9, 11, 12, 14, 15, 22, and 30 µm thickness (average of three values) were obtained at 10, 12, 14, 16, 18, 20, and 25 V, respectively. As the applied voltage increased, more charged particles were forced to move towards the working electrode and deposit there.

### 3.3. Deposition Yield

Deposition yield of coatings was calculated by using following formula:

Deposition yield = (∆W)/A (mg/cm^2^)

where ΔW is the change in the weight of substrates before and after coating, and ‘A’ represents the area of coated surfaces. Deposition yield was calculated for each parameter in triplicate, and the mean deposition yield (%) was plotted against applied voltages.

**Figure 3:**
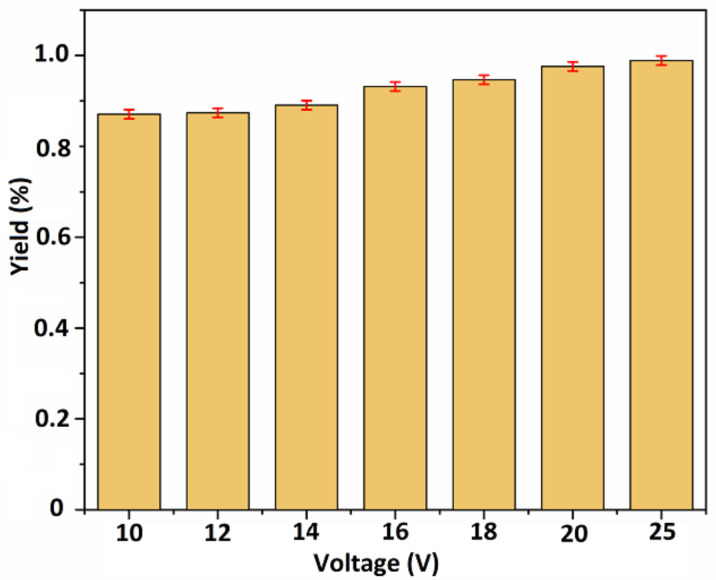
Deposition yield (%) graph with respect to the applied voltages. The highest deposition yield was achieved at higher voltage values, i.e., 18, 20, and 25 V, with slight standard deviations.

Figure 3 shows a consistent increase in the values of deposition yield (%) with the increase in voltage, which means that deposition yield is responsive towards the change in voltages. Thus, higher values of deposition yield were obtained at higher voltages (18, 20, and 25 V). The results of this study are in agreement with Hamaker’s law [41].

### 3.4. Adhesion Strength

**Figure 4:**
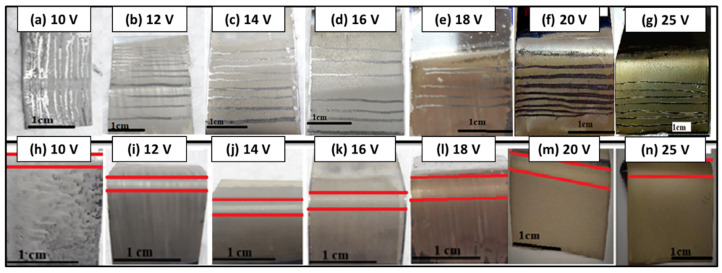
Pencil scratch test (**a**–**g**) and bend test results (**h**–**n**). Coatings deposited at 10 V and 12 V (**a**,**b**), detached even with softest pencil, i.e., grade 8B, coatings deposited at 14 V (**c**) detached at 5B, and coatings deposited at 16 V, 18 V (**d**,**e**), and 20 V (**f**) detached at 3B and 1B, respectively. The highest grade was achieved for the coating deposited at 25 V (**g**). The coatings deposited at 10 V (**h**), 12 V (**i**), and 14 V (**j**) showed delamination and micro-cracks around the bending site. Coatings deposited at 16 V (**k**) and 18 V (**l**) showed delamination around the edges of the bending site. The coatings deposited at 20 V (**m**) and 25 V (**n**) had no delamination or micro-cracks.

The adhesion strength of zein/Ag-Sr doped MBGN coatings deposited on SS was measured via pencil scratch test and bend test. Scratches were made on the surface of coated substrates by graphite pencils of different hardness grades (8B–2H), starting from the hardest grade, as shown in Figure 4a–g. Results of pencil scratch hardness tests were then recorded, as shown in Table 1. It was observed that coatings developed at higher voltages had higher hardness compared to those developed at lower voltages. The coating deposited at 25 V was graded ‘F’ according to the ASTM standard, which showed adequate adhesion strength between coating and substrate [43].

Adhesion strength between coatings and substrates was further examined via bend tests. The tests were performed manually by bending coated substrates at 180° with tweezers. Figure 4h–n show the images of the coated substrates taken after bend test. Upon bending, coatings deposited at 10, 12, 14, 16, and 18 V (Figure 4h–l) showed minor delamination around the edges at the bend site. No prominent crack or delamination was observed for coatings deposited at 20 and 25 V (Figure 4m,n), showing good adhesion strength between zein/Ag-Sr doped MBGN coatings and SS substrates [37,43]. These coatings were graded ‘4B’ according to the ASTM standard.

Morphological analysis of the coatings revealed that embedded Ag-Sr doped MBGNs in the zein matrix gain in homogeneity with the rise in deposition voltage, as shown in Figure 2o–u. Furthermore, the amount of Ag-Sr doped MBGNs within the coatings appear to rise proportionally with the increase in voltage, which could improve the mechanical strength of the coatings. Due to the increased amount of Ag-Sr doped MBGNs in the coatings, the adhesion strength of the coatings produced at higher voltages was higher than that of the coatings produced at lower voltages [44]. Moreover, the polymeric matrix (i.e., zein) may act as binder, holding the Ag-Sr doped MBGNs on the surface, giving a boost to the mechanical stability of the coatings.

### 3.5. Wettability Studies

Contact angle measurements were carried out to determine wettability of the coated substrates. Surface wettability of implanted materials is vital for successful implantation. When an implanted material enters into a physiological environment, it first interacts with body fluids, which further allow protein attachment. It is believed that cell adhesion and proliferation depend on the initial protein attachment [45]. If a surface allows initial protein attachment, it will subsequently allow cell adhesion and proliferation. Studies showed the surfaces with a contact angle in the range of 35–80° demonstrated good initial protein adsorption and osteoblast cell attachment [46,47]. 

**Figure 5:**
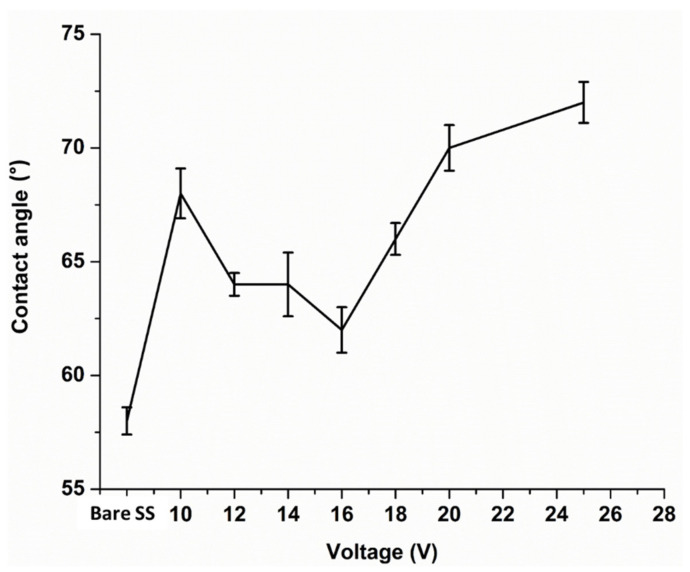
Contact angle graph for bare SS and coatings deposited at different voltages. Highest contact value was obtained for coating deposited at 25 V.

Contact angle tests were performed on bare SS and zein/Ag-Sr doped MBGN coated substrates. The results are plotted between contact angle (◦) and applied voltage (V) along with the standard deviation, as shown in Figure 5. The contact angle was measured five times for each type of coating. The bare SS showed an average contact angle of 58 ± 2°, which means that it is highly hydrophilic in nature. All the coated samples showed higher contact angle values as compared to the bare SS sample. Initially, a slight decrease in the values of contact angle was observed up until 16 V and, after that, values started to increase. Contact angle at 25 V was measured to be 72 ± 2°, which was the greatest among other coatings but perfectly within the suggested range for initial protein and osteoblast cell attachment.

The coatings exhibited lower hydrophilicity as compared to the bare SS. This effect is attributed to the presence of zein molecules. Zein is mostly composed of hydrophobic proteins [15], hence, the wetting behavior of coating shift towards hydrophobicity. However, the contact angles are still in the hydrophilic domain due to the enhanced deposition of Ag-Sr doped MBGNs at higher voltages. The MBGNs are majorly composed of a silica network. Silanol (Si-OH) groups present on the surface of silica make it intrinsically hydrophilic [48]. As the amount of zein deposition increases in the coating along with the voltage, it retains the hydrophilic character due to the presence of MBGNs [49].

### 3.6. Wear Studies

**Figure 6:**
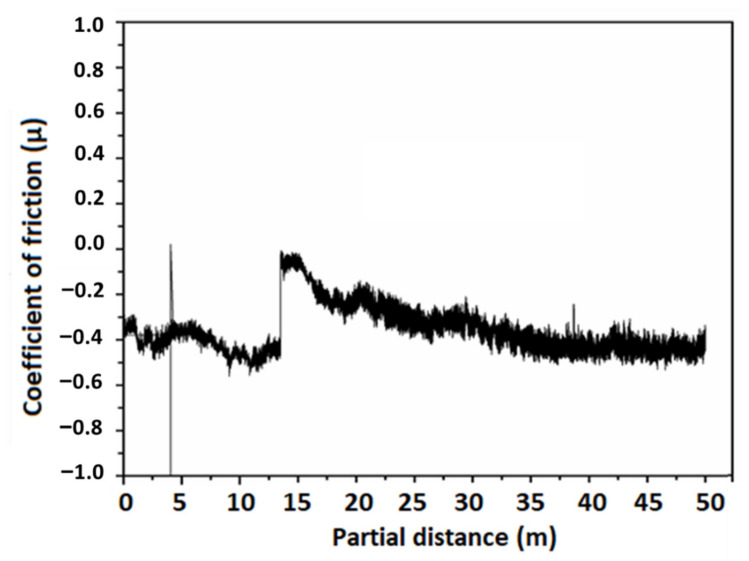
Graph between coefficient of friction (µ) and partial distance (m).

As discussed previously, the highest deposition yield and best adhesion results were found for the coating deposited at 25 V for 180 s. Thus, wear studies were conducted only for the coatings deposited at 25 V by using the ball-on-disk method on a tribometer. A steel ball indenter was rotated over the surface of a coating under a constant load of 1 N for a partial distance of 50 m. A graph was plotted between COF (µ) and partial distance (m), as shown in Figure 6. The graph shows that COF remained almost constant initially, however, there was an abrupt increase in COF around 15 m of partial distance. This behavior may be attributed to the removal of the transfer layer between contacting surfaces [50]. A transfer layer is formed due to the accumulation of the wear debris around the wear track, leading to abrasive wear. Later, COF lowered and became constant again throughout the partial distance, which means that the coating deposited at higher voltage displayed sufficient resistance to wear [51]. The average wear rate of zein/Ag-Sr doped MBGN coatings was calculated to be 0.179 mm**^3^**/Nm, which shows adequate wear resistance for a biocompatible implant [52].

The negative value of the friction co-efficient is due to the adhesive interaction between two contacting surfaces, i.e., the steel ball indenter and the zein/Ag-Sr doped MBGN coating. When the force is applied, the friction force initially increases. This may occur due to the strong adhesion of coating molecules (zein and Ag-Sr MBGNs) among themselves as compared to the adhesion between coating and substrate. The debris of coating starts to accumulate until the sliding distance of 15 m and then, upon persisting load, the layer is detached from the surface as a whole and not in the form of small patches. Hence, the presence of detached coating in the path of the wear track can increase the frictional force, which could lead to the negative COF value. The same phenomenon is noted by Thormann et al. [53] and Dedinaite et al. [54]. We also observed this behavior of negative COF in our previously published paper [55].

### 3.7. Corrosion Studies

**Figure 7:**
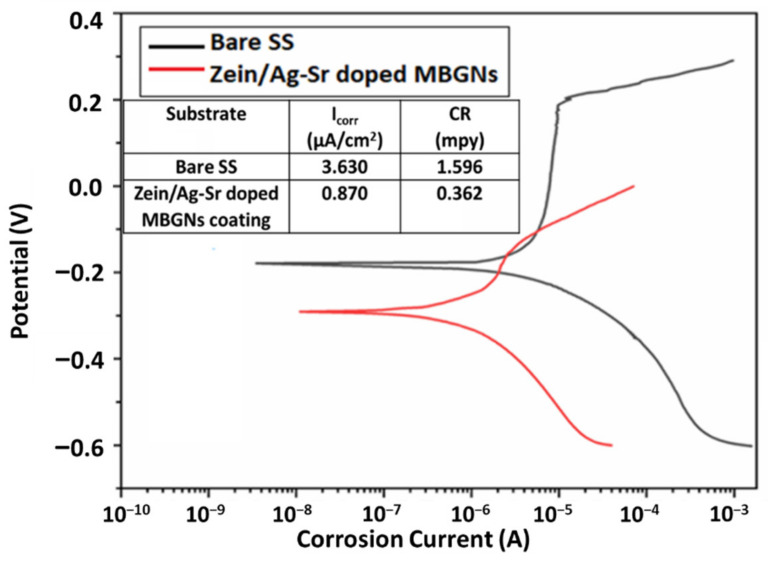
Potentiodynamic curves for bare and coated SS substrates.

To evaluate the corrosion performance of materials, corrosion current (I_Corr_) is measured in a relevant electrolyte; I_Corr_ is the amount of current flow while corrosion is taking place in an electrochemical cell. Metallic implants with low values of I_Corr_ and high corrosion potential are considered suitable for implantation [56]. Corrosion behavior of zein/Ag-Sr doped MBGN coatings deposited at 25 V was studied in SBF and compared with the corrosion behavior of bare SS with the help of potentiodynamic polarization curves, as shown in Figure 7. A potentiodynamic curve consists of anodic (upper) and cathodic (lower) curves. To better understand the corrosion behavior, the anodic curve is interpreted here. The graph shows the corrosion behavior of both bare SS and coated substrate. The anodic curve of bare SS shows an abrupt increase in the corrosion potential after a certain potential value. This abrupt increase indicates breakage of a passive layer that prevents corrosion, also reported in our previous study [57]. The breaking of this barrier layer results in the accelerated corrosion rate of the substrate material. However, the anodic curve of coated substrate shows no blunt increase in the values of corrosion potential.

The Tafel plot was fitted on these potentiodynamic curves with the help of Echem™ software. The I_Corr_ and corrosion rate (CR) for both bare and coated SS were calculated by the software. The values of I_Corr_ and CR for zein/Ag-Sr doped MBGN coatings deposited at 25 V were quite low as compared to those of bare SS substrates. Similar trends were observed in another study carried out by Ahmed et al. [16]. Zein/hydroxy apatite coating was deposited over 316L SS. The I_Corr_ was significantly lowered due to the presence of zein as compared to the bare substrate. It was concluded that zein coatings effectively increase the resistance against corrosion by fully covering the surface of the substrate. The Ag-Sr doped MBGNs were embedded inside the zein matrix, and both were strongly adhered to the substrate. Thus, in the present study, the corrosion resistance of the coated substrate was inferred to be higher in the physiological environment as compared to the bare SS substrates due to the presence of both zein and Ag-Sr doped MBGNs.

### 3.8. Biological Characterization

**Figure 8:**
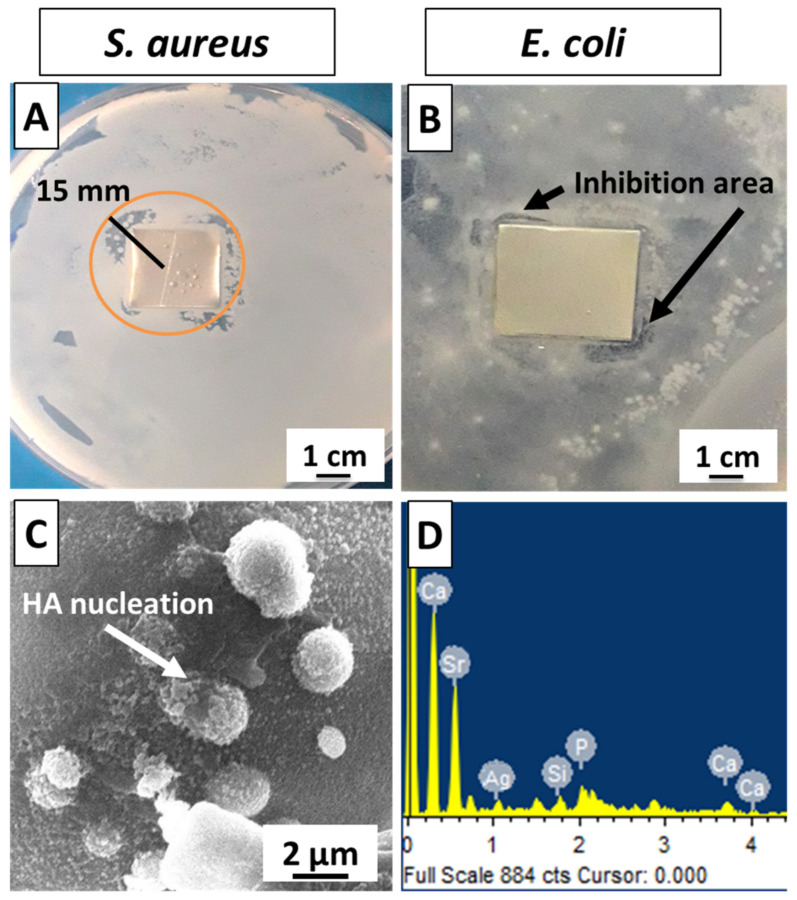
Antibacterial effect of zein/Ag-Sr doped MBGN coatings against (**A**) *S. aureus*, (**B**) *E. coli*, (**C**) HA nucleation starts in SBF at day 7; (**D**) EDS confirms presence of Ca and P on the surface of sample.

Zein/Ag-Sr doped MBGN coatings were tested against *S. aureus* and *E. coli* to determine the growth potential of bacteria. Figure 8A,B present the bacterial inhibition zones of tested samples. It was seen that a clear zone of inhibition (≈15 mm, measured from the widest side) formed in *S. aureus* (Figure 8A), whereas only a narrow inhibition area was observed around the two corners of the sample placed in *E. coli*, as marked in Figure 8B. This could have occurred due to the different cell membranes around the *S. aureus* and *E. coli*. It is reported that the outer membrane plays a significant role in protecting the bacteria from toxic materials [58]. The cell membrane around *E. coli* protects it from the antibacterial effect of the coating, whereas the absence of cell membranes around *S. aureus* renders it prone to coating effectiveness against it. It is expected that increasing the Ag amount in the MBGNs may increase the antibacterial efficiency of coating against *E. coli*; however, the exposure of Ag in the body above a safe level may cause cytotoxicity [23]. Therefore, optimizing the Ag quantity in MBGNs for efficiency against *E. coli* without exceeding its cytotoxic level could result in a very intriguing study topic.

Bioactivity is an important criterion in the selection of materials for tissue regeneration. In vitro bioactivity test in SBF was performed to detect the formation of an HA layer on the surface of the coating. A calcium phosphate-based HA layer is similar to natural bone mineral and facilitates the biological bonding between implant surface and surrounding tissues [55]. SEM analysis revealed the morphology of the coated sample after immersion in SBF for 7 days, as shown in Figure 8C. It was observed that HA crystals started to nucleate, and EDS (Figure 8D) also showed the presence of Ca and P in the spectra, which indicated the presence of calcium phosphate on the surface of coating. Hence, the zein/Ag-Sr doped MBGN coatings exhibited appreciable bioactivity.

## 4. Conclusions

In this work, zein/Ag-Sr doped MBGN coatings were developed on 316L SS by EPD at designated parameters of voltage and deposition time. The following conclusions were obtained at the end of the study.

- High deposition yield of the coatings was obtained at higher voltages, i.e., 25 V.
- Optical microscopic images showed uniform deposition of coatings on the surface of SS substrates (at optimum deposition parameters). SEM images illustrated the homogenous distribution of Ag-Sr doped MBGNs throughout the zein matrix along with the presence of spherical agglomerates, indicating good mechanical integration of zein/Ag-Sr doped MBGN coatings.
- Pencil scratch test results showed increased hardness of zein/Ag-Sr doped MBGN coatings deposited at 25 V, from which it was inferred that coatings developed at higher voltage showed improved adhesion strength. Furthermore, zein/Ag-Sr doped MBGN coatings exhibited good adhesion strength during bend tests.
- Zein/Ag-Sr doped MBGN coatings deposited at 25 V demonstrated good wettability properties (contact angle of 72 ± 2°), suitable for initial protein and subsequent osteoblast cell attachment.
- Moreover, zein/Ag-Sr doped MBGN coatings showed good wear and corrosion resistance as compared to that of bare SS substrates.
- Coatings exhibited good antibacterial and bioactive potential.

The above conclusions imply that zein/Ag-Sr doped MBGN coatings developed in this study via EPD at 25 V exhibited commendable mechanical and surface properties for biomedical applications.

## Figures and Tables

**Table 1:** Results of pencil scratch test.

Voltage (V)	Time (s)	Hardness Grade
10	180	8B
12	180	8B
14	180	5B
16	180	3B
18	180	3B
20	180	1B
25	180	F

## Data Availability

Data available upon request from the corresponding authors.
